# Substitution and Sudden Death

**Published:** 1904-03

**Authors:** 


### Substitution and Sudden Death.

THAT the use of depressant drugs bears direct relationship to sudden death has been demonstrated over and again by statistics. The *Journal of the American Medical Association,* January 16, 1904, in its editorial columns deals intelligently with this subject. In the city of New York the deaths from heart disease steadily increased during the years 1900, 1901, and 1902, in which the ratio of deaths of this kind in each thousand was 1.18, 1.31, and 1.34, respectively. The Journal comments on this subject as follows:

For the year 1903 there was a decrease in the number of deaths from this cause, so that the ratio was only 1.28 per thousand. There has been some discussion among sanitarians and public-health officials as to the reason for this decrease. A portion of the decrease has been ascribed definitely—and with considerable plausibility—to a certain cause. At the beginning of last year the board of health, suspecting that many prescriptions for phenacetin were being filled by druggists with acetanilid, or with a mixture of phenacetin and acetanilid, sent inspectors to obtain definite information on this matter. Altogether 373 samples of phenacetin were bought from the same number of drug stores in various parts of the city, phenacetin being specially asked for and in some instances even obtained, on a physician's prescription. Of the 373 samples, 58 were pure phenacetin; 315 were adulterated with cheaper drugs, mainly acetanilid, and in 267 cases containing more acetanilid than phenacetin; 32 samples were pure acetanilid. The commissioner made these facts public, and threatened to expose and prosecute all druggists who would hereafter be found committing this misdemeanor.

It is very interesting at least to find that a single year after the investigation and supposed consequent reform on the part of the dispensing pharmacists, there should be a slight reduction in the actual sudden death rate from heart disease, and that at a time when for many years there has been a constant increase in the death rate from this cause.

This question of the evil of depressant drugs is all the more interesting because of the freedom with which so-called headache powders, mainly composed of acetanilid and other heart depressants, are now so commonly bought and sold. Many women, and even men, think nothing of stepping into a drug store and asking for something for a headache. The headache powders that are dispensed to them so freely always contain acetanilid, and great harm is being done in this way. It is probable that a similar investigation in other cities of the country might also furnish instructive facts.

It will be observed from a careful examination of the foregoing that the ever present question of drug substitution receives a severe indictment, when many sudden deaths can be directly traced to its criminal employment. The law should be invoked and that speedily to regulate the dispensing of the class of drugs referred to, and to punish those who substitute one drug for another in physicians' prescriptions.

